# Is carbohydrate, protein, fat, and energy intake adequate among lactating women in Northwest Ethiopia?

**DOI:** 10.1186/s41043-026-01391-x

**Published:** 2026-07-19

**Authors:** Mahider Awoke Belay, Hanna Demelash Desyiblew, Samuel Dagne, Tadele Derbew Kassie, Mekuanent Asmare Yizengaw, Yonatan Menber

**Affiliations:** 1 Department of Public Health, College of Medicine and Health Science, Injibara University, Injibara, Ethiopia; 2 Department of Nutrition and Dietetics, School of Public Health, College of Medicine and Health Science, Bahir Dar University, Bahir Dar, Ethiopia; 3 Department of Public Health, College of Health Science, Debre Markos University, Debre Markos, Ethiopia; 4 Department of Anesthesia, School of Medicine, College of Medicine and Health Science, Injibara University, Injibara, Ethiopia

**Keywords:** Energy intake, Carbohydrate intake, Protein intake, Fat intake, Lactating women, Northwest Ethiopia

## Abstract

**Background:**

Maternal nutrition is an important factor in the health of lactating women and their children. Due to increased energy and nutrient needs during lactation, women in middle- and low-income nations are more nutritionally vulnerable than other women of reproductive age. Inadequate energy and macronutrient intake can also contribute to stunting, muscle wasting, edema, loss of bone density, anemia, and increased susceptibility to infections and nutritional deficiencies in women and babies. Some previous studies reported only the descriptive results of energy and macronutrient intake. But the current study assessed both the prevalence and associated factors. Therefore, this study aimed to assess energy and macronutrient intake inadequacy and its associated factors among lactating women in Bahir Dar City, Northwest Ethiopia.

**Methods:**

A study was conducted in Bahir Dar city from February 27 to March 21, 2021. A systematic random sampling technique was used to select 318 respondents. Data were collected by interviewer-administered, semi-structured questionnaires after the pilot survey had been completed. A single 24-hour multi-step dietary recall was used to collect the respondent’s dietary data. Data entry and analysis were performed using EpiData version 3.1 and SPSS version 24, respectively. Energy and macronutrient intake values were calculated from the ESHA Food Processor and the Ethiopian and World Food Composition Tables. The macronutrient intake was evaluated by the Acceptable Macronutrient Distribution Range (AMDR). Bivariable and multivariable binary logistic regression were used to declare the significant variables.

**Results:**

The prevalence of energy, carbohydrate, protein, and fat intake inadequacy among respondents was 52%, 66.2%, 17%, and 90.4%, respectively. The percentage of energy derived from carbohydrates, protein, and fat was 74%, 16%, and 10%, respectively. The median energy intake was 2416.8 kcal. Approximately 55%, 84%, and 90% of the respondents had carbohydrate, protein, and fat intakes above, within, and below the acceptable macronutrient distribution range, respectively. Not receiving nutrition education (AOR = 1.32, 95% CI (1.01, 2.83)) and being categorized as medium wealth (AOR = 2.04, 95% CI (1.03, 6.03)) were significantly associated with inadequate energy intake. Having a primary educational level (AOR = 2.56, 95% CI (1.07, 7.17)) and a secondary school educational level (AOR = 0.47, 95% CI (0.24, 0.93)) were associated with inadequacies in carbohydrate intake. Being a merchant (AOR = 2.01, 95% CI (1.12, 6.52)), daily laborer (AOR = 9.28, 95% CI (1.61, 43.65)), or private employee (AOR = 2.12, 95% CI (1.11, 8.07)) was associated with protein intake inadequacy. Due to the low prevalence outcome (< 10% adequacy) of fat intake, regression was not performed.

**Conclusion and recommendation:**

More than half of lactating women’s energy intake was lower than recommended. Most of the respondent’s carbohydrate, protein, and fat intakes were above, within, and below the acceptable macronutrient distribution range, respectively. Wealth index and nutrition education were associated with energy intake inadequacy. The educational status of the respondents’ husbands was associated with carbohydrate intake inadequacy, and the occupational status of the respondents and their husbands was associated with protein intake inadequacy. Therefore, lactating women should receive nutrition education and counseling to consume a diversified diet and enhance their dietary intake through healthcare practice.

## Introduction

Maternal nutrition is an important factor in the health of lactating women and their children. The mother’s health, the growth and health of the unborn child, and postpartum recovery all depend on the intake of nutrients [1]. The total amount of energy needed during lactation is the sum of the demands made before pregnancy plus the additional demands made by the requirement for sufficient milk secretion and production [2]. To make sufficient breast milk, additional energy is needed to promote postpartum recovery and maintain the optimal health of children and lactating women [3]. Adequate energy intake refers to the quantity of energy (calories) obtained from food that balances the body’s energy expenditure. This balance is essential for maintaining a healthy body weight and supporting physical activity, growth, and various physiological functions.

The amount of carbohydrates required to sustain normal blood glucose levels and function as the body’s main energy source is referred to as adequate carbohydrate intake. Adequate protein intake denotes the amount of protein needed to sustain body tissues, promote growth and repair, and provide essential amino acids required for various metabolic processes. The amount of dietary fat needed to deliver vital fatty acids, aid in the absorption of fat-soluble vitamins, and provide energy without raising health concerns is referred to as adequate fat intake [4]. Throughout lactation, a woman needs to consume an adequate amount of protein, carbohydrate, and fat to maintain her muscle mass and to provide adequate nutrition to her infant through breast milk to give enough nourishment, fostering the growth and development of infants [5]. Therefore, lactating women should adjust their dietary choices and their supplement intake to meet their needs.

Due to increased energy and nutrient needs during lactation, women in middle and low-income nations are more nutritionally vulnerable than other women of reproductive age [6, 7]. Research reports from Nepal [8], China [9], Indonesia [10], the Manawatu-Wanganui region of New Zealand [11], Umuahia of Nigeria [6], and Ethiopia [12, 13] revealed that the intake of carbohydrates, protein, and/or fat was insufficient. A study done in Bangladesh reported that energy and fat intake inadequacy was 74.4% and 98.3% respectively, among lactating women [14]. A systematic review and meta-analysis conducted in Indonesia and Malaysia revealed that energy and macronutrient intakes among pregnant women were below the recommended [15].

Many lactating women are not meeting the optimal nutrient intakes for both mothers and babies [16]. The poor nutritional status of lactating women stems from inadequate energy and macronutrient intake, alongside micronutrient malnutrition. This malnutrition can be attributed to insufficient vitamin and mineral density in their diets, low nutrient bioavailability, or heightened nutrient requirements due to factors such as infection [17]. Inadequate intake of energy and macronutrients in lactating women can also contribute to stunting, muscle wasting, edema, loss of bone density, anemia, decreased hormone synthesis (notably of growth and thyroid hormones and insulin), cardiovascular dysfunction, dermatitis, and increased susceptibility to infections and nutritional deficiencies in babies, putting them on a trajectory to potentially long-term negative consequences ranging from impaired growth, development, and learning readiness in early childhood to chronic diseases in adulthood [18].

As the world enters a new era of development, the Global Nutrition Report 2015 uses a host of facts, figures, and national experiences to urge the world to take action to end malnutrition in all its forms by 2030 [19]. Nutrition-sensitive and specific interventions and programs address some underlying causes of undernutrition, including poverty, food insecurity, poor health, and gender inequality. These efforts also encompass agriculture programs, social safety net initiatives, education programs, fortification, supplementation (of food or nutrients), and health and behavior change communication. These interventions aimed to enhance dietary habits and nutrition education and counseling, which are weapons for eradicating malnutrition throughout the world [20, 21]. To combat undernutrition, the Ethiopian government has launched numerous intervention initiatives. These include the National Nutrition Program (NNPII), the National Food and Nutrition Policy, the Health Extension Program (HEP) packages, micronutrient supplementation, and educating pregnant and breastfeeding women on dietary diversification [22, 23]. Despite those interventions, there was a high burden of lactating women’s undernutrition in Ethiopia [24–26]. Therefore, additional targeted studies, like those on energy and macronutrient intake inadequacy and its associated factors, are necessary in Ethiopia to design new implementation interventions more effectively. Most previous studies determined nutritional status by qualitative methods, but this study identified both the qualitative and quantitative measurements of dietary intake. This method makes this study unique and has a significant policy implication.

Some previous studies reported only the descriptive results of energy and macronutrient intake in Ethiopia, but no study was conducted in the current area. The current study identified both the prevalence and associated factors of energy and macronutrient intake inadequacy. However, the overall understanding of these inadequacies and their associated factors remains limited in Ethiopia. Therefore, this study aimed to assess energy and macronutrient intake inadequacy and its associated factors among lactating women in Bahir Dar city, Northwest Ethiopia. The findings of this study will provide valuable information for policymakers, governmental and non-governmental organizations, local administrators, and researchers to plan future circumstances regarding maternal nutrition intervention.

## Methods and materials

### Study setting and period

The study was conducted in Bahir Dar City, Northwest Ethiopia, which is the capital city of the Amhara region and geographically located between latitude 11°36′N and longitude 37°23′E. The city has six sub-cities and twenty-six kebeles, which are Fasilo sub-city (Fasilo 1, 2, 3, and 4); Belay Zeleke sub-city (Hagere Selam, Bisrat, and Kebele 7); Tana sub-city (Rasageze, Midre Genete, Shimbit, Hidasie, and Bata); Dagmawi Minilik sub-city (Adisamba, Midre Genete, Selamber, Marzeneb, and Finote); Atsie Tewodros sub-city (Teyima, Abay Ras, Ayer Tena, Adis Alem, and Maraki); and Gishabay sub-city (Abinet, Gion, Selamber, and Hidasie). It is also located 565 km away from Addis Ababa, the capital city of Ethiopia. According to the Bahir Dar Municipality Report 2019/2020, the total population was 312,410. Of them, approximately 53% were women [27]. The availability of a large number of food items in Bahir Dar city is one of the reasons to conduct this study. Some of the most common types of food found in Bahir Dar City include cereals and grains (nech teff injera, key teff injera, ambasha, boiled wheat nifro, porridge, pasta, steamed rice, and kolo), meat and milk (organ meat, yogurt, milk, cheese, and chicken meat), vegetables and fruits (cabbage, potato, tomato, green pepper, banana, and orange), and beverages (local tella, soft drinks, tea, and coffee). The study was conducted from February 27 to March 21, 2021.

### Study design and population

A community-based cross-sectional study design was employed. The source populations were all lactating women residing in Bahir Dar city, and the study populations were lactating women living in the selected kebeles of Bahir Dar city.

### Eligibility criteria

The study included all lactating women aged 18 to 49 who were breastfeeding their infants and had lived in Bahir Dar city for a minimum of six months. It excluded lactating women who had participated in ceremonies within the past 24 h. The participation of women in ceremonies such as marriage parties, feasts, christening celebrations, birthday parties, or graduation events can lead to an overestimation of their usual dietary intake.

### Sample size determination and sampling techniques

**Fig. 1:**
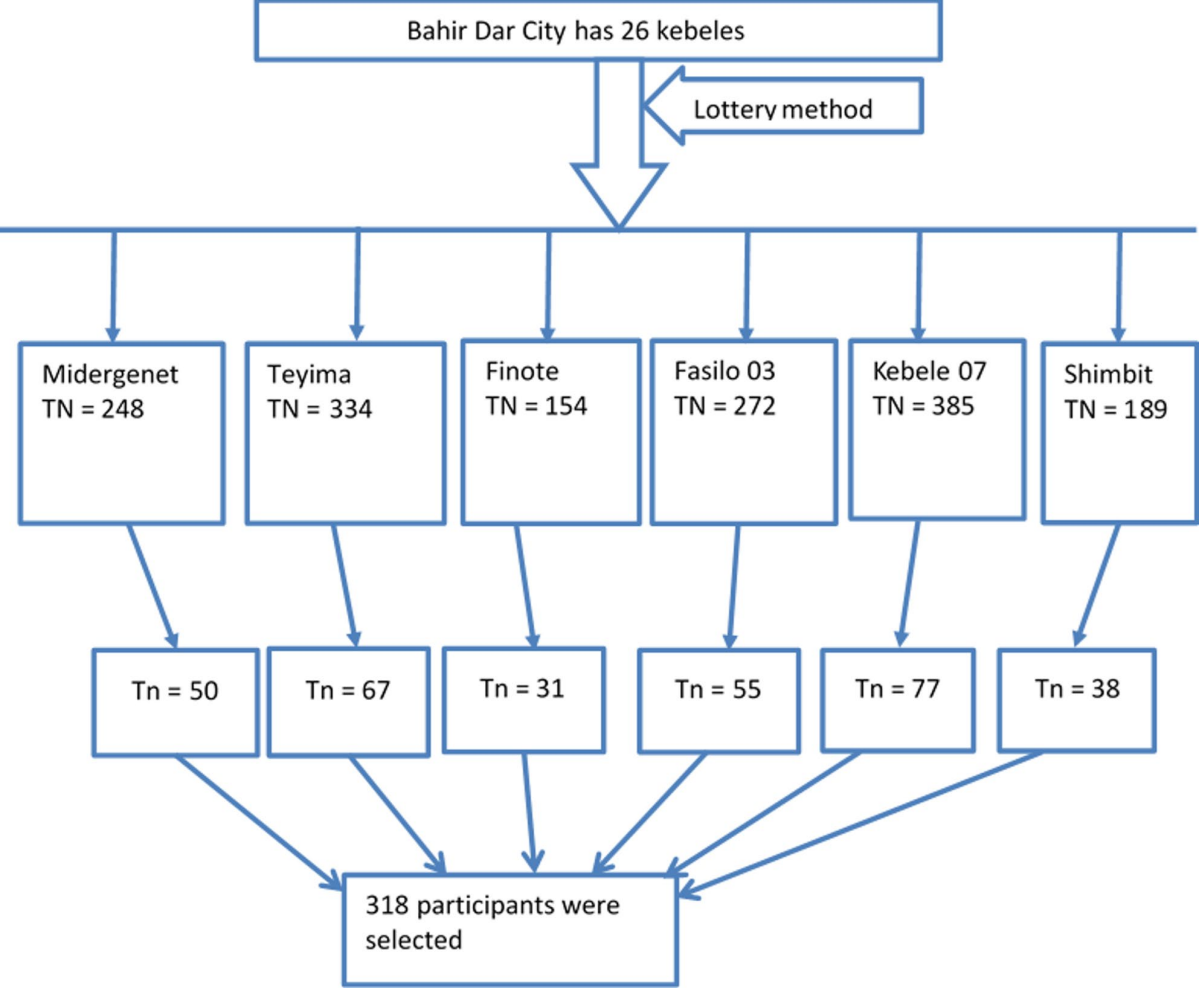
Schematic presentation of sampling technique for energy and macronutrient intake inadequacy among lactating women in Bahir Dar City, Northwest Ethiopia

The sample size was calculated by using Epi Info software, taking into account the assumptions of margin of error (4%), 95% confidence interval, and the prevalence of inadequate energy intake, which was found to be 14% among lactating women in Ethiopia [12]. The calculated sample size was 289. After adding a 10% non-response rate, the final sample size was 318. Among the 26 kebeles, six were selected using a lottery method, representing approximately 20% of the total kebeles. According to municipality statistics, there were 10,529 lactating women in Bahir Dar City. In the selected kebeles, there were 1,592 lactating women. Of them, 318 respondents were selected by using a systematic random sampling method. The value of ‘K’ is calculated from N/*n* = 1592/318 = 5, where N = study population and n = sample size. Then the respondents were proportionally allocated for each selected kebele using the formula of Tn = (n/N)*TN, where n = sample size, N = total number of lactating women in all selected kebeles (1592), TN = the total number of lactating women in each selected kebele, and Tn = the study participants in each kebele (Fig. 1).

### Operational Definitions

#### Adequate Intake

The actual intake of energy by the respondent is equal to or greater than the estimated energy requirement (EER) [28], and the macronutrient intake range should be within the Acceptable Macronutrient Distribution Range (AMDR).

#### Consumer

A lactating woman who consumed a certain food item or food group at least once in the day prior to the interview.

#### Portion Size

The amount of food or beverage consumed by lactating women at a particular time.

#### Kebele

The smallest administrative division of the federal government of Ethiopia [29].

### Data collection tools and procedures

The data collection tool (questionnaire) was written in English, then translated into the local language (Amharic), and then back-translated into English to ensure consistency. Data were collected by four trained public health officers and two BSc nurses and supervised by two public health officers. A standardized structured questionnaire was used to collect socio-demographic (age, religion, marital status, educational status, and occupational status of both the respondent and their husband) and economic variables (wealth index [30] and household food insecurity status [31]); nutritional knowledge-related; culture-related variables (food taboos) [32]; health-related (illness in the past month); and nutrition education service while she visited the health facility [33, 34]. Dietary data were assessed using the United Nations Food and Agriculture Organization (FAO) standardized tools [35].

**Food insecurity** was measured using the Household Food Insecurity Access Scale (HFIAS). The questionnaire consisted of nine occurrence questions representing the overall increasing severity of food insecurity (access) and nine “frequency of occurrence” questions that were asked as follow-up. Answer each onset question to determine how often the condition occurred over the previous 4 weeks (last month). Respondents were first asked a question about the occurrence, that is, whether the condition in question had occurred in the last four weeks (yes or no). If the respondent answered yes to the occurrence question, a question was asked about the frequency of occurrence in the last 4 weeks (rare (1 or 2 times), sometimes (3–10 times), or often (10 or more times)). HFIAS can be scored and classified as food secure or food insecure. Food secure: This category includes households where all members show no or minimal evidence of food insecurity (≤ 1 out of 27). Food insecurity includes households where all members feel anxious about running out of food or compromising the quality of foods they eat by choosing inexpensive options (2–27) out of 27 [31].

The household wealth index was based on the data collected in the household questionnaire. This questionnaire asks about the possession of various consumer goods by the household, such as a television and car, as well as details about the home, such as the type of flooring, the type of drinking water source, the restrooms, and other elements indicative of financial position. The resulting asset scores are generated by principal component analysis (PCA). Every non-dummy variable’s responses were divided into two categories. For the highest scores, codes were 1, and for the lowest scores, they were 0. Factor scores were generated in PCA using variables with a communality value larger than 0.5. Lastly, the wealth score was calculated using each household’s score on the first principal component. These scores are then used to establish the breakpoints that designate the five groups of wealth quintiles as poorest, poor, middle, rich, and richest [30].

The nutritional knowledge of the respondents was evaluated using ten multiple-choice and yes/no questions, with a maximum possible score of 24. The importance of carbohydrates, proteins, lipids, vitamins, and minerals; the sources of these nutrients; extra meals; and the function of iron and folic acid supplements were all questions used to evaluate it. A score of 1 was given to participants who correctly answered the knowledge-assessing questions, while a score of 0 was given to those who did not. The respondents were categorized as having poor, medium, and good nutritional knowledge based on their scores: those who answered less than 50% or scored fewer than 11 correct answers were classified as having poor nutritional knowledge; those who answered between 50% and 80% or scored between 12 and 19 correct answers were classified as having medium nutritional knowledge; and those who answered above 80% or scored 20 or more correct answers were classified as having good nutritional knowledge [32].

The nutrition education tools were adapted from the guidelines of National Nutrition Program II and Essential Nutrition Action (ENA). Validation was conducted after a pilot survey. During postnatal care (PNC) and child health visits, lactating women received nutrition education. Health care professionals offer nutrition education sessions that are brief, targeted, repeated, and practical. They also provide guidance on affordable and accessible food options, the frequency of meals, essential food inclusion for nutrition, strategies for promoting the health of both mother and child, and methods for enhancing the quantity and diversity of diets using local foods, all while adhering to the standardized national nutritional guidelines [33, 34].

For the dietary intake assessment, a single, multiple-pass, 24-hour recall method was used. Initially, common food items were recorded, along with photographs of household utensils. Subsequently, a code was assigned for data collection that corresponded to each household utensil. During the data collection, respondents were asked to list all food items they consumed over the previous 24 h, including any food or drink consumed outside their home. They were also instructed to indicate the portion sizes of these food items by using photographs of common household utensils for reference. The food items consumed by respondents, totaling 72, were measured using the corresponding household utensils to ensure standardization with the digital food weighing scale. The final measurements of these food items were recorded in grams [36]. The Ethiopian Food Composition Tables (EFCT) are compiled for various foods and beverages [37, 38]. The world food composition table (Kenya and Tanzania) was used to extract the values of specific food products and the incomplete nutrient values that were not included in the Ethiopian food composition table (e.g., boiled cow milk, honey, and soft drinks) [39, 40]. Dietary diversity score (DDS) was measured using a qualitative recall of all food categories consumed the day (24 h) preceding data collection. The FAO and FANTA III specified ten food groups to calculate the dietary diversity score. The ten food groups were (i) starchy staples, (ii) beans and peas, (iii) nuts and seeds, (iv) dairy products, (v) fresh foods (meat and fish), (vi) eggs, (vii) vitamin A-rich dark green leafy vegetables, (viii) other vitamin A-rich vegetables and fruits, (ix) other vegetables, and (x) other fruits. The DDS was calculated by totaling the number of food groups ingested each day. The mean DDS is referred to as the minimum dietary diversity score (MDDS). The women ate at least five food categories out of 10 considered as adequate DSS, and if the women ate fewer than 5 food groups, it was classified as inadequate DSS [41].

### Data quality control

Training was given to data collectors and supervisors that helped to maintain and improve the quality of the data. Before actual data collection, a pilot survey was conducted among 5% of the sample size from outside the study population. On-site data integrity checks were performed daily. A digital food scale was used to estimate the portion size of food items. The measurement device was calibrated to zero during standardization.

### Data management and analysis

The data were entered and analyzed using EpiData version 3.0 and IBM SPSS version 24.0, respectively. Data normality was checked using the Shapiro-Wilk test (*p* = 0.00). Normally distributed variables were presented as mean and standard deviation (mean, SD) (*p* ≥ 0.05), whereas skewed distributions were presented as median and interquartile range (*p* < 0.05). The binary logistic regression and descriptive analyses were conducted.

Energy intake was calculated from the ratio between the actual energy intake and the recommended energy intake (estimated energy requirement (EER)) of the respondents [6]. EER was calculated from the recommended dietary allowance (RDA) of energy for non-pregnant and non-lactating women plus the additional energy requirements during lactation for breast milk secretion [5]. The Institute of Medicine’s National Academies Press of America (IOM) was used to assess the intake of macronutrients based on the Acceptable Macronutrient Distribution Range (AMDR). This range includes carbohydrates (45–65%), protein (10–35%), and fat (20–35%) [42]. The percentage of energy from each macronutrient was calculated. The dietary intake data was entered by using the ESHA Food Processor Version 8.1 software. It was also used to determine macronutrient and calorie values of each food item. Finally, the results were exported to Excel and then imported into SPSS for analysis.

Both bivariable and multivariable binary logistic regression analyses were used to identify factors influencing inadequate energy intake among respondents. Variables observed in the bivariate binary logistic regression with *p* ≤ 0.25 were included in the multivariable binary logistic regression analysis. For the multivariable binary logistic regression models, a Hosmer-Lemeshow fit test was performed. Variables with *P* < 0.05 in the multivariable binary logistic regression analysis were considered statistically significant. The strength of the association between dependent and independent variables was expressed as an adjusted odds ratio (AOR). The final results were presented in the form of texts, figures, and tables.

## Result

### Sociodemographic and socioeconomic characteristics of the respondents

The study enrolled 311 lactating women, with a response rate of 97.8% and a median age of 29.0 ± 7.0 (median ± IQR). Most of the respondents, 258 (83%), were orthodox religious followers. Around 59% of the respondents’ husbands had college and above educational status. Approximately 40% of the respondents were housewives. In terms of marital status, 219 (93.6%) were married. The poorest and richest respondents were 49 (15.8%) and 99 (31.8%), respectively. Forty-one individuals (13.2%) reported having food insecurity (Table 1).

**Table 1:** Sociodemographic and socioeconomic characteristics of lactating women in Bahir Dar City, Northwest Ethiopia (*N* = 311)

Variables	Categories	Frequency (%)
Age	From 18 to 30 years of age	202(65.0)
	From 31 to 45 years of age	109(35.0)
Educational status of lactating women	Cannot read and write	35(11.3)
	Primary school	72(23.2)
	Secondary school	79(25.4)
	College and above	125(40.2)
Husband’s educational status	Cannot read and write	10(3.3)
	Primary school	55(18.3)
	Secondary school	59(19.6)
	College and above	177(58.8)
The religion of lactating women	Orthodox	258(83.0)
	Muslim	44(14.1)
	Protestant	9(2.9)
Occupational status of lactating women	Housewife	124(39.9)
	Government employee	83(26.7)
	Private employee	44(14.1)
	Merchant	48(15.4)
	Daily laborers and other workers	12(3.8)
Occupational status of the husband	Merchant	72(24.0)
	Government employee	129(43.0)
	Private employee	71(23.7)
	Daily laborers and other workers	28(9.3)
Marital status of lactating women	Married	291(93.6)
	Divorced	19(6.1)
	Widowed and other	1(0.3)
Postpartum days/months	45 days to 6 months	83(26.7)
	7–12 months 13–24 months	82(26.4) 146(46.9)
Number of children	< 5	302(97.1)
	≥ 5	9(2.9)
Family size	< 5	179(57.6)
	≥ 5	132(42.4)
Wealth quintiles	Poorest	49(15.8)
	Poor	72(23.2)
	Medium	38(12.2)
	Rich	53(17.0)
	Richest	99(31.8)
Household food insecurity status	Food-secure	270(86.8)
	Food-insecure	41(13.2)

### Health and cultural status of lactating women

Concerning the health status, 24 (6.3%) respondents were ill over the last month. Regarding the cultural status of food restriction, 25 (6.6%) respondents reported the presence of some food restriction. Alcohol (2.7%) was the most common food item that respondents avoided during breastfeeding. Furthermore, respondents indicated that rice (0.6%), lupine (gibito) (0.9%), chickpeas (0.9%), lemons (0.3%), corn (0.6%), and beans (0.6%) were avoided during lactation. Five (1.5%) respondents said that alcohol (0.6%), chickpeas (0.3%), and coffee (0.6%) were restricted by their family, pharmacist, husband, and self, respectively.

### Nutritional knowledge and nutrition education among lactating women

Nearly 70% of the respondents could define what a balanced diet is. Almost all, 99% of the respondents, answered the additional requirement for a meal during the lactation period. Approximately 66.2%, 58.2%, and 78% of the respondents correctly answered at least four sources of protein, carbohydrates, and fats, respectively. Around 52% of respondents answered at least three sources of vitamins and minerals. Regarding the importance of nutrients, 54.6% of respondents identified at least three benefits of protein, 44.9% for carbohydrates, 48% for fats, and 44% for vitamins and minerals. Additionally, 52.4% of respondents answered the importance of iron and folic acid supplementation. Approximately 34% of respondents had poor nutritional knowledge. Two hundred nine (67.2%) respondents did not receive nutrition education from health professionals (Table 2).

**Table 2:** Nutrition education and nutritional knowledge status of lactating women in Bahir Dar City, Northwest Ethiopia (*N* = 311)

Variables	Categories	Frequency (%)
Nutrition education received during the lactation period.	Yes	102(32.8)
	No	209(67.2)
Yes, they received nutrition education.	Sometimes	64(62.7)
	Usually	28(27.5)
	Always	10(9.8)
Learned the importance of iron-folic acid supplementation.	Yes	256(82.3)
	No	55(17.7)
Followed the hospital guidelines for nutrition and food during lactation.	Yes	13(4.2)
	No	298(95.8)
Nutritional knowledge status of lactating women	Poor	107(34.4)
	Medium	111(35.6)
	Good	93(30.0)

### Energy and macronutrient intake among lactating women

The median energy intake was 2416.8 kcal ± 934.3 (median ± IQR). Approximately 52% (95% CI 46.4, 57.8) of respondents had inadequate energy intake. The median intake of carbohydrates, protein, and fat among the respondents was 443.4 g, 109.6 g, and 28.3 g, respectively. Two hundred fifty-eight (83%) of the respondents had protein intake within the AMDR, and 169 (54.3%) had carbohydrate intake greater than the AMDR. A high proportion (87.8%) of lactating women’s fat intake was below the AMDR (Table 3). The prevalence of carbohydrate, protein, and fat intake inadequacy was 66.2% (95% CI; 60.7, 71.5), 17% (95% CI; 13.0, 21.7), and 90.4% (95% CI; 86.5, 93.4), respectively.

**Table 3:** Macronutrient intake and its relation to AMDR among lactating women in Bahir Dar City, Northwest Ethiopia (*N* = 311)

Nutrients	Median/IQR (Q1-Q3)	*N* (%) below AMDR	*N* (%) within AMDR	*N* (%) above AMDR	RDA/AMDR/EER
Energy (kcals) Carbohydrate (g)	2416.8 ± 934.3 443.4 (169.8)	37 (11.9)	105 (33.8)	169 (54.3)	2550 kcal 292.5^x^ (45–65%)
Protein (g)	109.6 (68.1)	44 (14.1)	258 (83)	9 (2.9)	71 (10–35%)
Fat (g)	28.3 (26.9)	273 (87.8)	30 (9.6)	8 (2.6)	57.8^x^ (20–35%)

^“X”^ means the dietary reference nutrient intake of carbohydrate (45%) and fat (20%) was calculated from the assumption of AMDR to the requirement of energy to make its contribution based on its minimum requirement

**Fig. 2:**
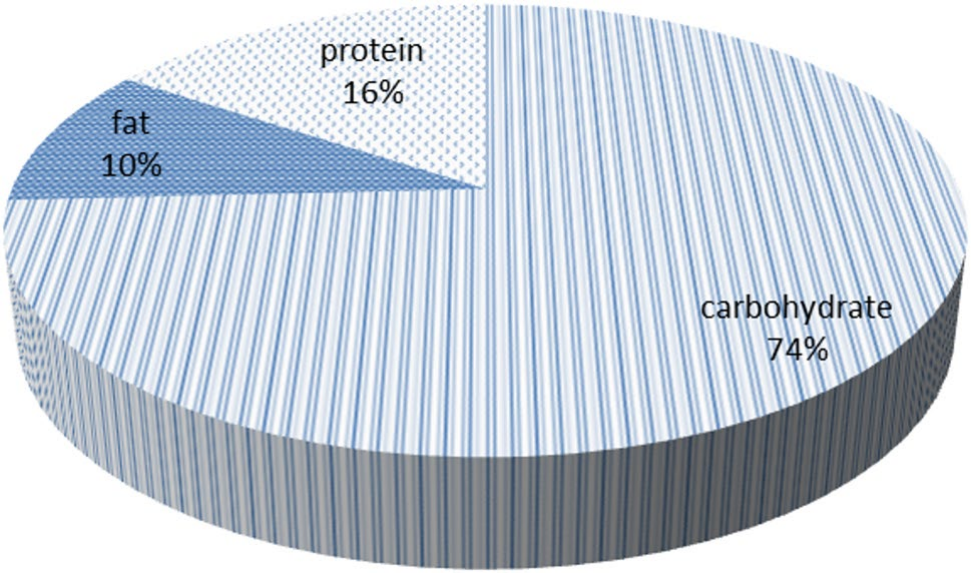
Percent of energy contributions from carbohydrate, protein, and fat among lactating women in Bahir Dar City, Northwest Ethiopia, 2021 (*N* = 311)

The median energy contribution from the three macronutrients was as follows: Fat intake was below the recommended guidelines for balanced nutrition (< 20% of energy intake (95% CI 7.0, 14.1)), carbohydrate intake was above the maximum percentage (> 65% of energy intake (95% CI 68.9, 79.0)), and protein intake was within the acceptable range (10–35% (95% CI 11.4, 20.7)) (Fig. 2).

### Energy and macronutrient intake in relation to the postpartum period of lactating women

Approximately 42% of the lactating women within the postpartum period of 13 to 24 months had fat intake below the AMDR. The median intakes of carbohydrates, protein, and fat for 45 days to 6 months of the postpartum period were 462 ± (239), 117 ± (84), and 23 ± (27), respectively. Among respondents who had a postpartum period of 45 days to 6 months, energy intake inadequacy was 12%. Most of the respondents in the postpartum period of 13 to 24 months had protein intake within the AMDR (38%) (Table 4).

**Table 4:** The relationships between energy, macronutrient intake, and the postpartum periods in relation to AMDR among lactating women in Bahir Dar City, Northwest Ethiopia (*N* = 311)

Macronutrient intake	Postpartum periods			
		45 days to 6 months	7 to 12 months	13 to 24 months
Carbohydrate (N (%))	Below AMDR	6 (1.93)	15 (4.82)	16 (5.14)
	Within AMDR	28 (9.0)	23 (7.40)	54 (17.36)
	Above AMDR	49 (15.76)	44 (14.15)	76 (24.44)
Protein (N (%))	Below AMDR	13 (4.18)	12 (3.85)	19 (6.11)
	Within AMDR	72 (23.15)	67 (21.54)	119 (38.26)
	Above AMDR	2 (0.64)	3 (0.97)	4 (1.29)
Fat (N (%)) Energy (N (%))	Below AMDR	75 (24.12)	67 (21.55)	131 (42.12)
	Within AMDR	7 (2.25)	11 (3.54)	12 (3.86)
	Above AMDR Adequate Inadequate	2 (0.64) 45 (14.47) 38 (12.22)	5 (1.60) 40 (12.86) 42 (13.50)	1 (0.32) 64 (20.58) 82 (26.37)

### The relationship between energy intake and dietary diversity status of lactating women

Approximately 63% of the respondents had inadequate dietary diversity status. Within 24 h, all respondents ate starchy foods. The majority of respondents (95.2%) consumed tomato and onion over the previous 24 h. Nearly 94% of the respondents had not yet eaten nuts and seeds. Only 5 respondents ate other fruits in the past 24 h (Table 5). From the results of person correlation, there was no significant linear association between dietary diversity status and energy intake (*r* = 0.04, p-value = 0.53).

**Table 5:** The dietary diversity status of lactating women in Bahir Dar City, Northwest Ethiopia (*N* = 311)

S. no.	Food Groups	Response	Frequency (%)
1.	Starchy staples	Yes	311(100.0)
2.	Beans and peas	Yes	235(75.6)
3.	Nuts and seeds	Yes	19(6.1)
4.	Dairy products	Yes	62(19.9)
5.	Fresh foods (meat and fish)	Yes	118(37.9)
6.	Eggs	Yes	60(19.3)
7.	Vitamin A-rich dark green leafy vegetables	Yes	108(34.7)
8.	Other vitamin A-rich vegetables and fruits	Yes	101(32.5)
9.	Other vegetables	Yes	296(95.2)
10.	Other fruits	Yes	5(1.6)
Dietary diversity score (DDS)		Inadequate DDS	197(63.3)
		Adequate DDS	114(36.7)

### Factors associated with energy intake inadequacy among lactating women

The following variables had p-values ≤ 0.25 in the bivariable binary logistic regression: lactating women’s educational and occupational status, husbands’ educational and occupational status, family size, nutrition education, postpartum months, and wealth index. In the multivariable analysis, the wealth index and received nutrition education were associated with energy intake inadequacy. Respondents who did not receive nutrition education were 1.32 times more likely to develop energy intake inadequacy than the corresponding [AOR = 1.32, 95% CI (1.01, 2.83)]. According to the wealth index, being in the medium was 2.04 times more likely to develop energy intake inadequacy than being the richest [AOR = 2.04, 95% CI (1.03, 6.03)] (Table 6).

**Table 6:** Bivariable and multivariable binary logistic regression analyses of energy intake inadequacy among lactating women **in** Bahir Dar City, Northwest Ethiopia (*N* = 311)

Variables	Categories	Energy Intake (N)			
		Inadequate	Adequate	COR (95% CI)	AOR (95% CI)
Educational status of lactating women	Cannot read and write	18	17	1.01(0.48, 2.14)	0.79(0.46, 3.59)
	Primary school	33	39	0.81(0.45, 1.44)	0.80 (0.45, 1.69)
	Secondary school	47	32	1.40(0.79, 2.48)*	1.38 (0.88, 2.94)
	College and above	64	61	1	1
Educational status of the husband	Cannot read and write	6	4	1.30 (0.35, 4.75)	1.03(0.21, 5.03)
	Primary school	22	31	0.61(0.33, 1.14)*	0.51(0.22, 1.24)
	Secondary school	32	27	1.02(0.57, 1.85)	1.01 (0.51, 1.99)
	College and above	95	82	1	1
Occupational status of lactating women	Housewife	57	67	1	1
	Government employee	43	40	1.26 (0.72, 2.21)	1.01(0.55, 2.21)
	Private employee	25	19	1.55 (0.77, 3.09)*	1.53 (0.69, 3.58)
	Merchant	29	19	1.79 (0.91, 3.53)*	1.65(0.75, 3.66)
	Daily laborers and other workers	8	4	2.35(0.67, 8.21)*	2.01 (0.41, 10.62)
Husband’s occupational status	Merchant	43	29	1	1
	Government employee	65	64	0.69(0.38, 1.23)*	0.68(0.32, 1.44)
	Private employee	35	36	0.66(0.34, 1.27)*	0.62(0.34, 1.54)
	Daily laborers and other workers	13	15	0.58(0.24, 1.41)*	0.57 (0.19, 1.85)
Family size	< 5	84	95	1	1
	≥ 5	78	54	1.63(1.04, 2.57)**	1.62 (0.94, 2.77)
Nutrition education	Yes	48	54	1	1
	No	114	95	1.35 (0.84, 2.17)*	1.32 (1.01, 2.83)**
Postpartum months	45 days to 6 months	38	45	1	1
	7 to 24 months	124	104	1.41(0.85, 2.34)*	0.62(0.34, 1.09)
Wealth Index	Poorest	21	28	0.55(0.28, 1.10)*	0.52(0.34, 1.51)
	Poor	30	42	0.53(0.29, 0.97)**	0.50 (0.22, 0.1.08)
	Medium	28	10	2.06(0.90, 4.71)*	2.04 (1.03, 6.03)^**^
	Rich	26	27	0.71(0.36, 1.39)	0.68(0.34, 1.36)
	Richest	57	42	1	1

* significant at p-value ≤ 0.25; ** significant at p-value < 0.05; unmarked = not significant; 1 = reference group; COR = crude odds ratio; AOR = adjusted odds ratio; CI = confidence interval

### Factors associated with carbohydrate intake inadequacy among lactating women

In the bivariable binary logistic regression, the variables that had p-values ≤ 0.25 were marital status, educational status of the husband, nutritional knowledge, occupational status of the women, and wealth index. In the multivariable analysis, the educational status of the husband was statistically significant. The odds of carbohydrate intake inadequacy were higher for those with only a primary education than for those with a college degree or higher [AOR = 2.56, (95% CI; 1.07, 7.17)]. Compared to college and above, those with only a secondary education had 53% lower odds of carbohydrate intake inadequacy [AOR = 0.47, (95% CI: 0.24, 0.93)] (Table 7).

**Table 7:** Bivariable and multivariable binary logistic regression analysis of carbohydrate intake inadequacy among lactating women in Bahir Dar City, Northwest Ethiopia (*N* = 311)

Variables	Categories	Carbohydrate intake (N)			
		Inadequate	Adequate	COR (95% CI)	AOR (95% CI)
Educational status of the husband	Cannot read and write	4	6	0.31(0.08, 1.14)*	0.22(0.05, 1.07)*
	Primary school	45	8	2.60 (1.15, 5.89)**	2.56 (1.07, 7.17)**
	Secondary school	31	28	0.51(0.28, 0.94)**	0.47(0.24, 0.93)**
	College and above	121	56	1	1
Occupational status of lactating women	Housewife	91	33	1	1
	Government employee	53	30	0.64(0.35, 1.17)*	0.57(0.28, 1.14)*
	Private employee	26	18	0.52(0.26, 1.08)*	0.51(0.22, 1.18)*
	Merchant	29	19	0.55(0.27, 1.12)*	0.47(0.21, 1.03)*
	Daily laborers	7	5	0.51(0.15, 1.71)	0.49(0.08, 3.21)
Nutritional knowledge level	Poor	74	33	0.93(0.51, 1.71)	0.92(0.36, 3.52)
	Medium	67	45	0.62(0.34, 1.11)*	0.61(0.32, 1.18)*
	Good	65	27	1	1
Marital status	Married	197	95	2.30(0.91, 5.86)*	0.39(0.07, 2.19)
	Divorced	9	10	1	1
Wealth Index	Poorest	34	15	0.94(0.45, 1.98)	0.65(0.21, 1.99)
	Poor	43	29	0.61(0.32, 1.17)*	0.58(0.28, 1.20)*
	Medium	24	14	0.71(0.32, 1.56)	0.62(0.27, 1.45)
	Rich	35	18	0.81(0.39, 1.65)	0.79(0.31, 1.93)
	Richest	70	29	1	1

* significant at p-value ≤ 0.25; ** significant at p-value < 0.05; unmarked = not significant; 1 = reference group; COR = crude odds ratio; AOR = adjusted odds ratio; CI = confidence interval

### Factors associated with protein intake inadequacy among lactating women

In the bivariable binary logistic regression, the following factors had a P value of ≤ 0.25: wealth index, nutritional knowledge, marital status, educational and occupational status of both lactating women and their husbands, and food security. In the multivariable binary logistic regression analysis, protein intake inadequacy was significantly associated with the occupational status of the respondents and their husbands. The odds of having inadequate protein intake were twice [AOR = 2.01, 95% CI (1.12, 6.52)] and nine times [AOR = 9.28, 95% CI (1.61, 43.65)] higher for merchants and daily laborers, respectively, than for housewives. Husbands with private employees were more likely than merchants to have protein intake inadequacy [AOR = 2.12, 95% CI (1.11, 8.07)] (Table 8). Due to the low prevalence outcome (< 10% adequacy) of fat intake, regression was not done.

**Table 8:** Bivariable and multivariable binary logistic regression analysis of protein intake inadequacy among lactating women in Bahir Dar City, Northwest Ethiopia (*N* = 311)

Variables	Categories	Protein intake (N)			
		Inadequate	Adequate	COR (95% CI)	AOR (95% CI)
Educational status of lactating women	Cannot read and write	7	28	1.83(0.68, 4.93)*	0.58(0.11, 3.26)
	Primary school	15	57	1.93(0.88, 4.23)*	1.87(0.71, 4.19)*
	Secondary school	16	63	1.86(0.86, 4.02)*	1.83(0.64, 3.76)*
	College and above	15	110	1	1
Educational status of the husband	Cannot read and write	3	7	2.38(0.58, 9.78)*	1.74(0.20, 15.43)
	Primary school	10	43	1.29(0.58, 2.88)	0.70(0.19, 2.63)
	Secondary school	8	51	0.87(0.37, 2.04)	0.50(0.17, 1.49)*
	College and above	27	150	1	1
Occupational status of lactating women	Housewife	16	108	1	1
	Government employee	12	71	1.14 (0.51, 2.56)	1.08(0.42, 3.68)
	Private employee	7	37	1.28 (0.49, 3.35)	0.89(0.30, 2.67)
	Merchant	11	37	2.01(0.86, 4.71)*	2.01 (1.12, 6.52)**
	Daily laborers	7	5	9.45 (2.68, 33.38)**	9.28 (1.61, 43.65)**
Husband’s occupational status	Merchant	8	64	1	1
	Government employee	21	108	1.56(0.65, 3.72)	1.44(0.58, 5.62)*
	Private employee	15	56	2.14(0.85, 5.43)*	2.12 (1.11, 8.07)**
	Daily laborers	6	22	2.18 (0.68, 6.99)	1.43(0.27, 7.66)
Marital status	Married	46	246	1	1
	Divorced	7	12	3.12 (1.17, 8.34)**	1.96(0.17, 22.43)
Nutritional knowledge	Poor	22	85	2.39 (1.04, 5.48)**	1.49 (0.48, 4.66)
	Medium	22	90	2.25(0.98, 5.18)*	2.09(0.83, 5.27)*
	Good	9	83	1	1
Food security status	Insecure	12	29	2.31 (1.09, 4.90)**	1.40(0.32, 6.22)
	Secure	41	229	1	1
Wealth Index	Poorest	12	37	2.60 (1.05, 6.41)**	1.72(0.38, 7.90)
	Poor	12	60	1.60(0.66, 3.86)	1.37 (0.49, 3.83)
	Medium	8	30	2.13(0.78,5.80)*	1.94(0.64, 5.89)*
	Rich	10	43	1.86(0.33, 4.72)*	1.76(0.64, 4.82)
	Richest	11	88	1	1

* significant at p-value ≤ 0.25; ** significant at p-value < 0.05; unmarked = not significant; 1 = reference group; COR = crude odds ratio; AOR = adjusted odds ratio; CI = confidence interval

## Discussion

The median intakes of energy, carbohydrate, protein, and fat among the respondents were 2416.8 kcal, 443.4 g, 109.6 g, and 28.3 g, respectively. Nutrition education and the wealth index were the two significant factors associated with energy intake inadequacy among respondents. The educational status of the respondent’s husband was significantly associated with carbohydrate intake inadequacy, and the occupational status of the lactating women and their husbands was associated with protein intake inadequacy.

In line with the current study, similar findings were reported in the Tigray region [12], Niger [43], Nepal [8], China [17], Khorramabad in Iran [44], Malaysia and Indonesia [15], and Europe [45]. This finding implies that inadequate intake of energy might be due to low socioeconomic status, poor nutritional knowledge, food restriction, or large family sample size.

Conversely, the current finding showed a lower median energy intake compared to the research done in Indonesia [46] and the Philippines [47]. This discrepancy in results could be attributed to the sample size and the primary dietary source, as the current study area has a high consumption of plant-based meals, particularly cereals and legumes. The smaller sample size may lead to a larger value and lack its representativeness, and the sample size for Indonesia and the Philippines was 111 and 70, respectively.

The median carbohydrate intake was 443.4 g, which exceeds 52% of the recommendation. This finding aligns with previous reports conducted in Nigeria (77.7%) [6], Nepal (80%) [8], and China (62.7%) [9]. In contrast, another study indicated that in China, carbohydrate intake levels were 33% to 49% below the recommended nutrient intake [48]. It is not surprising that lactating women had a higher carbohydrate intake. Since cereals are the most significant staple foods in this research area, high cereal eating probably leads to high carbohydrate intake. These results also showed that the respondents mostly rely on staple plant-based diets. Consuming large amounts of carbohydrates may raise the risk of non-communicable disease.

Furthermore, the median protein intake was 109.6 g, exceeding the recommended level by 54%. This finding aligns with another study, which indicated that protein intake was higher by 31% to 53% than the recommended levels [48]. However, some studies reported lower results [9, 44]. This difference may be due to the fact that protein sources are predominantly plant-based (cereals and legumes) and widely consumed in the study area. The health implication of this finding is that high protein intake may lead to overweight and obesity, which could potentially increase the risk of chronic diseases.

The median intake of fat among lactating women in the present study was 28.3 g, which was lower by 51% than the recommended amount. This finding corresponds to the results of the study done in Nepal (29 g/d) [8]. Although this result was lower than the two study findings in China, it was the lower Chinese RNI (57.8 g) [9, 48]. Differences in results are due to socioeconomic conditions and low intake of fatty source foods such as fish, seeds, nuts, and other vitamin A-rich vegetables and fruits (such as avocados) in the study area. Inadequate intake of fat may lead to deficiency of essential fatty acids and fat-soluble vitamins and defects in metabolic regulation.

The recommended level of energy distribution is 45–65% for carbohydrates, 10–35% for protein, and 20–35% for fat. In this study, the median contribution of energy from carbohydrates was observed to be 74%, which exceeds the recommended level. Protein accounted for 16%, falling within the recommended distribution range. In contrast, fat contributed only 10%, which is below the recommended level. Another study, conducted in Shanghai, China, said that the median contribution of carbohydrates to the energy ratio was 45%, at or slightly below the recommended level; protein was 20%, within the recommended level; and fat was 34%, near or above the recommended level [49]. Moreover, the median percent contribution of energy from carbohydrates was 70%, slightly above the upper limit of the AMDR, and those of protein and fat were at or slightly below the lower limit of the AMDR in Niger (10% and 20%, respectively) [50]. The average percentage of energy derived from protein, fat, and carbohydrates in the results of the Nepal study was 11%, 13%, and 76%, respectively [8]. This difference may be due to the higher consumption of cereal-based foods in the study area, which are the main food staples consumed in developing countries, including Ethiopia, and the higher consumption of fatty foods such as seafood in developed countries. Likewise, the differences may be due to the socioeconomic, sociodemographic, and seasonal characteristics of the variation. The new findings of our study imply that high consumption of carbohydrates (74% above AMDR) may lead to weight gain, obesity, type 2 diabetes, hypertension, and other adverse health outcomes associated with increased adiposity. Conversely, low consumption of fat may result in liver dysfunction due to mitochondrial dysfunction and increased susceptibility to infections [51]. Adequate consumption of protein is essential for optimal bone development and growth, reduces appetite and hunger levels, increases muscle mass and strength, boosts metabolism, increases fat burning, lowers blood pressure, and helps maintain weight loss and well-being [52].

One of the significant factors associated with energy intake inadequacy was nutrition education. The likelihood of energy intake inadequacy was significantly higher among those who did not get nutrition education than among those who did. Qualitative research supports this conclusion [3]. The impact of nutrition education on women’s knowledge, attitudes, and skills will increase their understanding of how to change maternal feeding habits to provide nutrition, which has positive effects on health; choose appropriate and safe food ingredients; and use good food to prevent nutritional disorders [53]. There was a strong correlation found between increased nutrition education and higher use of dark (whole grain) bread, decreased consumption of meat, and energy drinks sweetened with sweets [54]. Nutrition education is an essential tool for enhancing nutrient intake, as it emphasizes self-efficacy. This focus helps individuals gain the confidence needed to cook, prepare, and select healthy foods. Lactating women are advised to ensure an adequate dietary intake that includes two additional meals per day. This should focus on dietary diversity and quantity by using local staple foods, particularly those rich in iron and vitamin A. It is also important to use iodized salt and to drink plenty of fluids. These recommendations are provided by healthcare professionals during PNC or child health visits [34].

Furthermore, the wealth index was associated with inadequate energy intake. Respondents in the medium wealth index were more times likely to have inadequate energy intake than the richest. In developing countries, socioeconomic status influences food intake and consumption. The economic standing of a household is a sign of access to sufficient food supplies, healthcare services, and access to better water sources and sanitation facilities, all of which are important determinants of maternal nutritional status [55]. Individuals with medium income levels may experience greater energy intake inadequacy due to income instability, as they often depend on small businesses, government jobs, or informal work. When the prices of local staple foods increase, individuals’ purchasing ability may decrease; as a result, they may reduce the frequency of their meals or the portion size. People with low incomes may rely on social safety nets or food aid, whereas those with medium incomes lack external support, such as social safety nets or food aid, which may heighten the risk of inadequate food intake.

The present study showed that the educational status of the respondent’s husband was associated with carbohydrate intake inadequacy. Compared to those with college degrees and beyond, those with only a primary education level were more likely to develop carbohydrate intake inadequacy. However, compared to those with a college degree or higher, those with only a secondary education level were less likely to have carbohydrate intake inadequacy. Respondents with lower levels of education may also have poorer socioeconomic status, which restricts their ability to buy nutritious foods, and may have lower understanding of balanced diets and healthy foods. Higher educated individuals, on the other hand, are more likely to seek nutritional recommendations from health professionals or scientific sources and may have a solid understanding of balanced diets and healthy eating.

The respondent’s occupational status was associated with protein intake inadequacy. The risk of protein intake inadequacy among merchants and daily laborers was more times greater as compared to housewives. Daily laborer women find it difficult to fulfill the basic needs of their lives since they have low socioeconomic status and strive to purchase diversified food items on a daily basis. Conversely, compared to housewife women, merchant women might not be challenged with the issue of a financial crisis but rather with the difficulty of not having access to prepared and cooked meals more often at work. The respondent’s husband’s occupational status was the significant factor associated with protein intake inadequacy. Husbands who worked as private employees were more likely to have protein intake inadequacy as compared to merchants. This might be due to the private employees’ low salaries, inability to meet their basic needs, and inability to purchase a large assortment of fruits and vegetables.

## Conclusions and recommendations

The energy intake of lactating women was lower than the recommended estimated energy requirement. The contribution of energy from carbohydrates was higher, while that from fat was lower than the AMDR. But the energy ratio of proteins was within the AMDR. The majority of the respondents had an inadequate dietary diversity score. There was no linear correlation between energy intake and dietary diversity score. In the multivariable binary logistic regression analysis, nutrition education and the wealth index were significantly associated with energy intake inadequacy. The educational status of the respondent’s husband was significantly associated with carbohydrate intake inadequacy, and the occupational status of the lactating women and their husbands was associated with protein intake inadequacy. Due to the low prevalence outcome of fat intake, regression was not performed. Therefore, it is crucial to provide nutrition education on appropriate dietary intake for the respondents and their husbands. Lactating women should receive advice to consume a diversified diet and enhance their dietary intake through educational healthcare practices. Organizations responsible for maternal and child health should take proactive measures to support lactating women living in impoverished and even medium socioeconomic conditions. It is also advised that lactating women increase their daily intake of fatty food sources and eat diversified food.

### Strengths and limitations of the study

Attaining detailed information on foods and beverages consumed in the past 24-hour period, providing a quantitative estimate of the respondent’s food consumption, and recording the actual intake of energy and macronutrients were some of the strengths of this study. Additionally, the limitations of this study include the undetermined cause-and-effect relationship due to its cross-sectional effect; the lack of assessment of usual dietary intake due to the study’s single-day nature; social desirability bias; portion size estimation biases; the dependence on the non-Ethiopian food composition table; and the fact that the contribution of each food item to energy, carbohydrate, protein, and fat was not assessed due to time constraints, and over- and under reporters for energy intake were not calculated. To minimize the potential for recall and social desirability bias, a multiple-pass 24-hour recall method was employed, and participants received a detailed explanation of the study objectives prior to the commencement of data collection.

## Data Availability

All relevant data are available in the manuscript without restriction.

## Abbreviations

AMDR: Acceptable Macronutrient Distribution Range
EER: Estimated Energy Requirement
FAO: Food and Agriculture Organization of the United Nations
HFIAS: Household Food Insecurity Access Scale; IQR: Interquartile Range
